# Effect of A Polyphenol-Rich *Canarium album* Extract on the Composition of the Gut Microbiota of Mice Fed a High-Fat Diet

**DOI:** 10.3390/molecules23092188

**Published:** 2018-08-30

**Authors:** Ning-Ning Zhang, Wen-Hui Guo, Han Hu, A-Rong Zhou, Qing-Pei Liu, Bao-Dong Zheng, Shao-Xiao Zeng

**Affiliations:** College of Food Science, Fujian Agriculture and Forestry University, Fuzhou 350002, China; znnfst@163.com (N.-N.Z.); gwhfst@163.com (W.-H.G.); hhh3661@163.com (H.H.); zar0306@163.com (A.-R.Z.); jasonlau2013@163.com (Q.-P.L.)

**Keywords:** *Canarium album*, polyphenol, gut microbiota, high-fat diet

## Abstract

This study investigated the influence of *Canarium album* extract (CAext) on intestinal microbiota composition of mice fed a high-fat diet (HFD). Kun Ming (KM) mice were fed either a normal chow diet or a HFD for six weeks. At the seventh week, HFD-fed mice were gavaged daily with saline, or a different dose of CAext for four weeks, respectively. Then, the composition of the gut microbiota was analyzed by high-throughput sequencing technology. Analysis of fecal microbial populations, grouped by phyla, showed significant increases of *Firmicutes* and *Verrucomicrobia*, but a decrease of *Bacteroidetes* in all CAext-fed mice. Particularly, CAext gavage in a low dose or a medium dose caused a significant increase in the proportion of *Akkermansia*. These findings suggested that CAext can alter the gut microbiota composition of HFD-fed mice, and had a potential prebiotic effects on *Akkermansia*.

## 1. Introduction

The mammalian gut is inhabited by a vast number of microorganisms [1]. It has been well recognized that a balanced gut microbiota composition confers benefits to the host, whereas gut microbiota imbalance may lead to various metabolic diseases [2]. As the composition of diet has significant effects on gut microbiota, the interest in food-based strategies able to modulate the gut microbiota composition, and probably their functional effects, has rapidly increased in recent years [3]. It has been reported that plant-derived polysaccharide foods increase colonic bifidobacterial numbers [4], whereas a long-term diet rich in saturated fat, such as high-fat diet (HFD), increases the proportion of *Firmicutes* to *Bacteroidetes*, which is associated with obesity-induced metabolic diseases [5,6,7].

Polyphenols are poorly absorbed by the small intestine [8], and as much as 90% of polyphenols arrive at the large intestine [9], where interactions may happen between polyphenols and gut microbiota. Polyphenols can be converted to bioactive compounds that may exert physiological effects by the gut microbiota, while the composition of microbiota can be modified by polyphenols [10]. *Canarium album* is a type of polyphenol-rich fruit with a total phenolic content of 1174.0–1799.6 mg gallic acid equivalents/100 g fresh weight [11]. Its high polyphenol content is related to hepatoprotective [12], antioxidant [13], anti-inflammatory [14], and antiviral [15] effects. We previously found that polyphenol-rich *Canarium album* extract (CAext) restrains excessive lipid accumulation induced by oleic acid in hepatocarcinoma cells [16]. However, whether the pharmacological effects of polyphenols are attributed to its interactions with gut microbiota is not fully understood [10]. In light of this, the aim of the present study was to investigate the in vivo effects of CAext on the composition of intestinal microbiota of HFD-feeding mice.

## 2. Results

To understand the effect of CAext administration on the composition of gut microbiota, the gut microbiota composition was analyzed by Miseq sequencing on the V3–V4 region of 16S rRNA of bacteria. A total of 189,910 valid sequences and 146,325 operational taxonomic units (OTUs) were obtained after quality control, and the average length of each sequence was 442 bp.

Microbial phylotype richness was estimated by Chao and Ace, and the diversity of the bacterial community was revealed by Shannon [17]. As shown in Table 1, there was a significant overall decrease in the richness and the diversity of gut microbiota in the mice fed by HFD (*p* < 0.05). The NC group had the highest microbial phylotype richness and diversity in all groups, whereas the CAext groups had the lowest richness and diversity in all groups.

**Figure 1:**
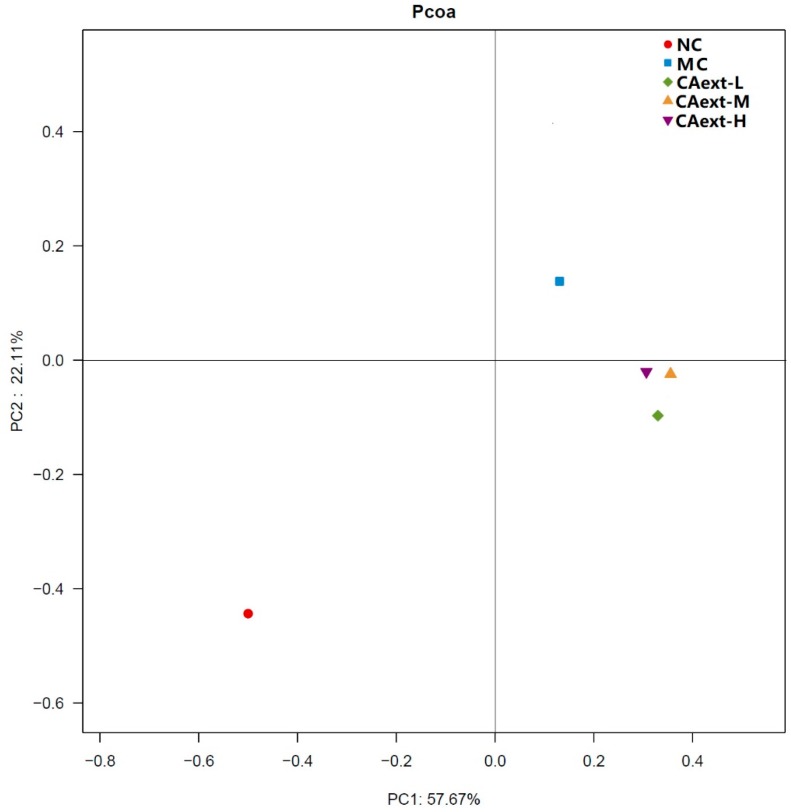
Principal coordinate analysis (PCoA) plots based on the OTU abundance of gut microbiota of each mouse. The closer the spatial distribution between spots, the greater the bacterial similarity among different groups. NC, normal control, mice were fed with a normal chow and intragastrically administered 20 mL/kg saline; MC, model control, mice were fed with a high-fat feed and intragastrically administered 20 mL/kg saline; CAext-L, CAext-M, CAext-H mice were fed with a high-fat feed and intragastrically administered 10 mg/(kg·d), 15 mg/(kg·d), and 20 mg/(kg·d) *Canarium album* extract (CAext), respectively.

**Figure 2:**
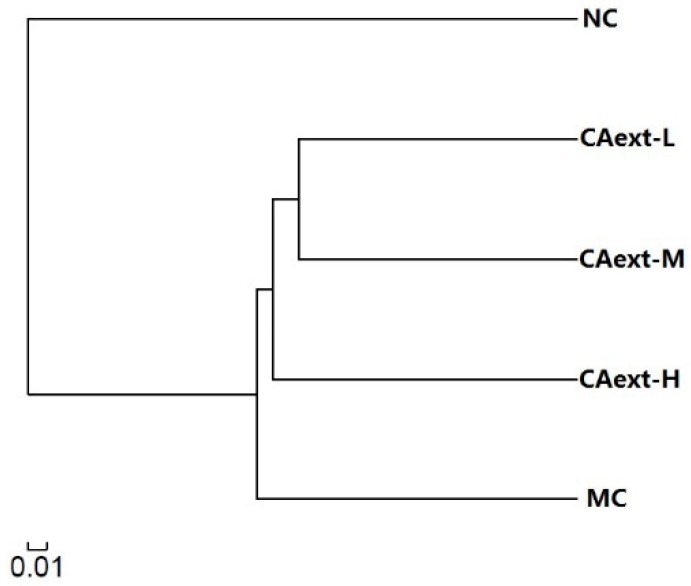
Clustering analysis dendrogram of gut microbiota based on distances among different groups. NC, normal control, mice were fed with a normal chow and intragastrically administered 20 mL/kg saline; MC, model control, mice were fed with a high-fat feed and intragastrically administered 20 mL/kg saline; CAext-L, CAext-M, CAext-H mice were fed with a high-fat feed and intragastrically administered 10 mg/(kg·d), 15 mg/(kg·d), and 20 mg/(kg·d) *Canarium album* extract (CAext), respectively.

Principal coordinate analysis (PCoA) based on the OTUs’ abundance of different groups was performed to evaluate the similarities and differences among groups (Figure 1). The five groups were separated as two clusters along PC1 (57.67%), suggesting that there were significant differences in the dominant bacterial population among the groups. The MC group was clearly separated along PC2 axis, indicating that CAext administration had a substantial effect on the gut microbial composition of HFD-fed mice. Cluster analysis of gut microbiota among different groups also had a similar result (Figure 2).

**Figure 3:**
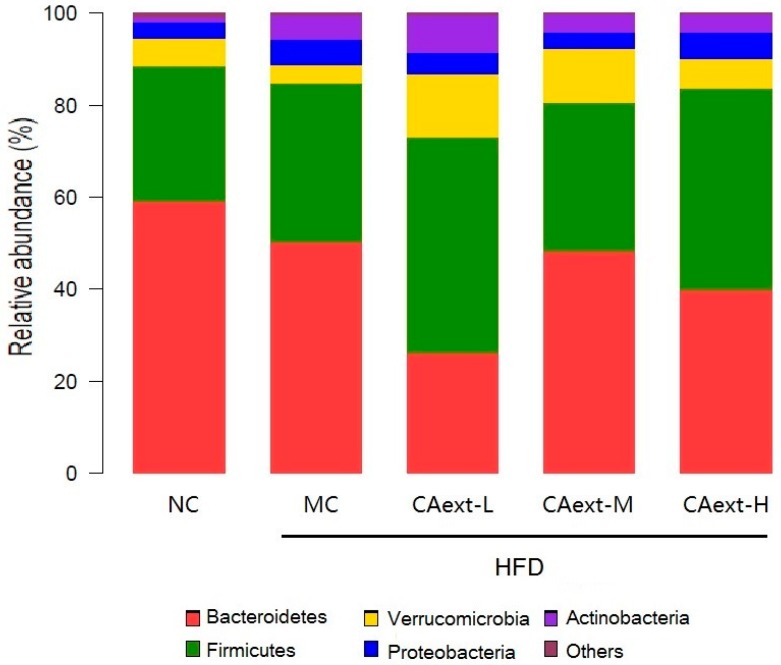
Relative abundance at the phylum level of gut microbiota among different groups. Other phyla refer to a taxa with a relative abundance ≤1% in any sample. NC, normal control, mice were fed with a normal chow and intragastrically administered 20 mL/kg saline; MC, model control, mice were fed with a high-fat feed and intragastrically administered 20 mL/kg saline; CAext-L, CAext-M, CAext-H mice were fed with a high-fat feed and intragastrically administered 10 mg/(kg·d), 15 mg/(kg·d), and 20 mg/(kg·d) *Canarium album* extract (CAext), respectively.

Analysis of relative bacterial abundance at the phylum level revealed the major differences of the dominant bacterial population among groups (Figure 3). Compared to the NC group, the relative abundance of *Bacteroidetes* was significantly reduced, whereas that of *Firmicutes* increased in all HFD-fed groups, especially in CAext-L and CAext-H group (*p* < 0.05). The *Firmicutes* to *Bacteroidetes* (F/B) ratio were respectively 0.50, 0.69, 1.79, 0.67, 1.09 in NC, MC, CAext-L, CAext-M, CAext-H group. Furthermore, the relative abundance of *Verrucomicrobia* was statistically higher in CAext-L and CAext-M groups compared with the NC or MC group (*p* < 0.05).

**Figure 4:**
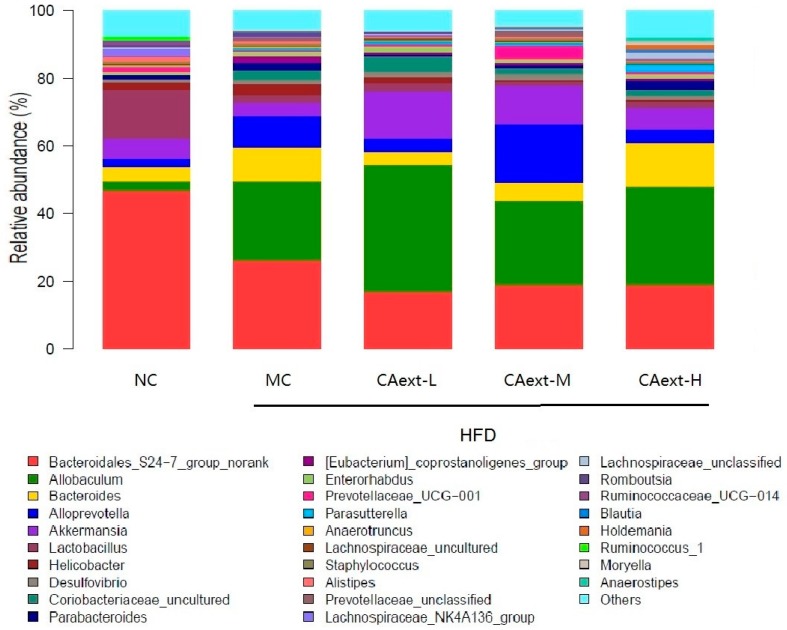
Relative abundance at the genus level of gut microbiota among different groups. Other genera refer to a taxa with a relative abundance ≤1% in any sample. NC, normal control, mice were fed with a normal chow and intragastrically administered 20 mL/kg saline; MC, model control, mice were fed with a high-fat feed and intragastrically administered 20 mL/kg saline; CAext-L, CAext-M, CAext-H mice were fed with a high-fat feed and intragastrically administered 10 mg/(kg·d), 15 mg/(kg·d), and 20 mg/(kg·d) *Canarium album* extract (CAext), respectively.

At the genus level (Figure 4), consumption of HFD statistically reduced the relative abundance of genus *Bacteroidales_S24-7*, which might be related to the reduction in the *Bacteroidetes* phylum, while the increase of the *Firmicutes* phylum might be attributed to the significant increase in the *Allobaculum* genus of all HFD-fed groups (*p* < 0.05). Compared to the MC group, CAext administration significantly decreased the abundance of *Bacteroidales_S24-7* (*p* < 0.05). However, no significant difference was obtained for the abundance of *Allobaculum* between MC and CAext groups (*p* > 0.05). As shown in Figure 4, CAext-L and CAext-M administration significantly increased the relative abundance of *Akkermansia* within the *Verrucomicrobia* phylum compared to the NC group or MC group (13.89 ± 3.78% in CAext-L, 11.68 ± 4.12% in CAext-M vs. 6.01 ± 3.92% in NC, 4.37 ± 3.03% in MC; *p* < 0.05).

## 3. Discussion

HFD feeding can alter the diversity and composition of intestinal microbiota [18], and CAext administration further reshapes the gut microbiota of HFD-fed mice. Using high-throughput sequencing, we found significant changes in gut microbiota composition of different treatment groups. The richness and the diversity of gut microbiota significantly decreased in all HFD-fed groups (Table 1). Similarly, Xia et al. [19] investigated the effect of whole grain Qingke on intestinal microbiota of rats under HFD, and found that the alpha diversity indices were lower in the HFD group than in the other groups, and ascribed this decrease to dominant bacteria restraining others’ growth. *Bacteroidetes* and *Firmicutes* are two major phyla dominant in most mammalian gut microbiota. Several studies have shown that obese subjects have a lesser proportion of *Bacteroidetes*, a higher proportion of *Firmicutes*, and a higher F/B ratio compared to normal subjects [8,20]. Our results found that HFD loading induced a drop in *Bacteroidetes* and an increase in *Firmicutes* (Figure 3). Particularly, CAext administration was associated with a striking increase in the F/B ratio (0.50 in NC group vs. 1.79, 0.67, 1.09 in CAext-L, CAext-M, and CAext-H, respectively). Similar results were found by Anhe et al. [21], indicating that *Firmicutes* significantly increased and *Bacteroidetes* significantly decreased after polyphenol-rich cranberry extract treatment. Queipo-Ortuno et al. [22] found that both the concentration of *Firmicutes* and *Bacteroidetes* were significantly increased after a polyphenol-rich red wine period.

In the present study, we found that the relative abundance of *Verrucomicrobia* was statistically higher in CAext group compared with NC or MC group (Figure 3). This increasing of the proportion of *Verrucomicrobia* attributed to a significant increase of genus *Akkermansia* (Figure 4). Several studies have shown that different sources of dietary polyphenols increased the relative abundance of *Akkermansia*. Axling et al. [23] reported that green tea polyphenol increased the proportion of *Akkermansia*. Kemperman et al. [24] also found that complex polyphenols from black tea increase the relative abundance of *Akkermansia*. *Akkermansia*-like microorganisms are widely distributed in the intestines of the animals and human beings [25], and at least eight different species of the *Akkermansia* genus colonize the intestines of humans [26]. *Akkermansia* is a Gram-negative, strict anaerobe and mucin-degrading bacterium [27]. The beneficial effects of *Akkermansia muciniphina* have been extensively studied [28,29,30]. Multiple findings suggested that the improvement of metabolic disorders were associated with the increase of the *Akkermansia* population [31]. It has also been proved that *Akkermansia* administration has probiotic effects, which are associated with the ability of *Akkermansia* to preserve the mucus layer thickness [25], therefore reducing gut permeability [29], systemic lipopolysaccharide levels [21], host adiposity [32], and inflammatory markers [29].

Because of the tissue anatomical and physiological differences between species [33], animal models of HFD cannot fully replicate the complexity of human pathological conditions [34,35]. The results of this study may only provide a reference for studying the effect of CAext on the human microbial ecosystem. Another limitation is whether *Akkermansia*-related beneficial effects are sufficient to prevent the negative metabolic phenotype associated with major modifications in the proportions of *Firmicutes* and *Bacteroidetes*. Further research should be performed to clarify the effects of CAext on *Akkermansia*, and the mechanisms by which CAext or polyphenols reshape the gut microbiota with benefits to the host.

## 4. Materials and Methods

### 4.1. Preparation of CAext

CAext were prepared according to the method of Liu et al. [16]. Total polyphenol content was measured using the method of Giampieri et al. [36]. Total polyphenols content of CAext was 465.35 ± 13.67 mg/g.

### 4.2. Animal Experiments

All animal procedures were approved by the animal ethics committee of Fujian University of Traditional Chinese Medicine (Ethic Approve No. FJATCM-IAEC 2016012). Forty male KM mice (20 ± 2 g), purchased from The Fujian Wus Laboratory Animal Co., Ltd. (Fujian, China), were housed in a room with a temperature of 24 ± 2 °C, a humidity of 55 ± 5%, and a 12:12 h light:dark cycle (8:00 am–8:00 pm).

The mice were fed a normal standard chow diet during their one week acclimatization period. After that, eight mice were randomly selected as the normal control group (NC), fed with a normal standard chow diet, and the rest of the thirty-two mice were fed with HFD for six weeks. According to Li et al. [37], the composition of the normal standard chow diet and the HFD with small changes are shown in Table 2. At the seventh week, the mice fed with HFD were randomly divided into the following four groups: model control group (MC, *n* = 8), low dose of CAext-treated group (CAext-L, *n* = 8), medium dose of CAext-treated group (CAext-M, *n* = 8), and high dose of CAext-treated group (CAext-H, *n* = 8). The mice in MC and NC groups were treated with a dose of 20 mL/kg saline once a day by gavage. The mice of the CAext-L, CAext-M, or CAext-H groups were orally administered a dose of 10 mg/kg, 15 mg/kg, or 20 mg/kg CAext once a day, respectively. After four weeks of treatment with the above solution, all mice were sacrificed. The feces were collected and stored at −80 °C for further analysis.

### 4.3. Fecal DNA Extraction

Total DNA from fecal samples of eight mice per group was extracted using a TIANamp Stool DNA Kit (DP328, Tiangen Biotech (Bejing) Co., Ltd., Beijing, China). The concentration and the purity of DNA were determined using the NanoDrop 2000 spectrophotometer (Thermo Scientific, Waltham, MA, USA). The integrity of the extracted DNA was measured on 1.0% agarose gel.

### 4.4. Fecal Microbiota Analysis

The extracted DNA was used as a template to amplify the V3-V4 region of the bacteria 16S rRNA gene (forward primer for V3 5′-ACTCCTACGGGAGGCAGCA-3′, reverse primer for V4 5′-GGACTACHVGGGTWTCTAAT-3′). The amplification program was as follows: initial denaturation at 95 °C for 3 min, amplification for 27 cycles of denaturation at 95 °C for 30 s, annealing at 55 °C for 30 s, and extension at 72 °C for 45 s, then extension at 72 °C for 10 min.

Sequencing was performed according to the protocol of Illumina Miseq platforms at Shanghai Majorbio Bio-Pharm Technoloy Co., Ltd., Shanghai, China.

To obtain high-quality reads, the raw reads were quality filtered using QIIME software (Version 1.9.0, http://qiime.org). The chimeric sequences were identified and removed with the UCHIME algorithm (UCHIME Algorithm, http://www.drive5.com/usearch/manual/uchime_algo.html). The remaining sequences with 97% identity were clustered into the same operational taxonomic units (OTUs) using Usearch software (Version 7.1, http://drive5.com/usearch/). The representative sequences of each OUT were assigned to RDP classifier (Version 2.2, http://rdp.cme.msu.edu/classifier). Alpha diversity index based on the OTUs was conducted by Mothur software (Version 1.30.0, http://www.mothur.org). All the statistical analyses of the sequenced data were performed by using the R software package (Version 3.2.3, http://www.R-project.org).

### 4.5. Statistical Analysis

Results are represented as mean ± SD. Statistical analysis was performed through one-way analysis of variance (ANOVA) using IBM SPSS 22.0 (IBM, Armonk, NY, USA), *p* < 0.05 was considered statistically significant. 

## 5. Conclusions

In conclusion, administration of polyphenol-rich CAext altered the composition of the gut microbiota with an increase in the relative abundance of *Firmicutes* and *Verrucomicrobia*, along with a decrease in *Bacteroidetes*. Furthermore, CAext-L and CAext-M greatly increased the population of the *Akkermansia*. Further studies will elucidate the prebiotic effect of CAext on the gut microbiota.

## Figures and Tables

**Table 1:** Effect of CAext on the phylotype richness and the diversity of gut microbiota.

	NC	HFD			
		MC	CAext-L	CAext-M	CAext-H
Ace	298.38 ± 11.63 ^a^	266.80 ± 11.07 ^b,c^	272.74 ± 14.05 ^b^	251.69 ± 13.70 ^c^	222.70 ± 10.44 ^d^
Chao1	304.65 ± 19.48 ^a^	269.87 ± 15.92 ^b^	279.94 ± 22.79 ^a,b^	254.45 ± 18.83 ^b^	223.33 ± 13.17 ^c^
Shannon	4.19 ± 0.02 ^a^	3.73 ± 0.02 ^b^	3.07 ± 0.02 ^c^	3.20 ± 0.02 ^d^	3.54 ± 0.02 ^e^

Data are presented as mean ± SD. NC, normal control, mice were fed with a normal chow and intragastrically administered 20 mL/kg saline; MC, model control, mice were fed with a high-fat feed and intragastrically administered 20 mL/kg saline; CAext-L, CAext-M, CAext-H mice were fed with a high-fat feed and intragastrically administered 10 mg/(kg·d), 15 mg/(kg·d), and 20 mg/(kg·d) *Canarium album* extract (CAext), respectively. Different letters indicate significant difference between treatment groups (*p* < 0.05).

**Table 2:** The composition of the normal standard chow diet and the HFD.

Ingredients (g/100 g)	Normal Standard Chow Diet	HFD
Corn starch	30	26.3
Wheat bran	25	21.9
Fish meal	5	4.4
Soy bean flour	20	17.5
Wheat flour	16	14.0
Yeast powder	1	0.9
Bone meal	2	1.8
Salt	1	0.9
Cholesterol	-	2
Lard oil	-	10
Sodium deoxycholate	-	0.3
